# Effect of abdominoplasty with transverse plication on rectus diastasis

**DOI:** 10.1016/j.jpra.2026.01.047

**Published:** 2026-01-31

**Authors:** Jordi Descarrega, Joan Fontdevila, Marta Suñer, Ferran Escrigas, Héctor Oyonate, Erica Segura, Albert Castro, Karam Yousef

**Affiliations:** a Plastic Surgery Department, Hospital Clínic de Barcelona Carrer de Villarroel, Facultat de Medicina, Campus Clínic Carrer de Casanova, Barcelona 08036, Spain; b Plastic Surgery Department, Hospital Clínic de Barcelona Carrer de Villarroel, Barcelona 08036, Spain; c Universitat de Barcelona, Facultat de Medicina, Campus Clínic Carrer de Casanova, Barcelona 08036, Spain

**Keywords:** Abdominoplasty, Transverse plication, Longitudinal plication, Diastasis recti, Bulging

## Abstract

Transverse plication (TP) abdominoplasty has gained popularity in recent years, as it is the keystone step of the TULUA procedure. This technique can achieve excellent contour results and reduce complications. However, the effect of TP on diastasis recti abdominis (DRA) remains uncertain. This study is the first specific research designed to investigate the effect of TP on the inter-recti distance (IRD). Two different strategies were defined for this purpose. The first is a prospective self-controlled case series study with ultrasound evaluation on 16 consecutive post-bariatric patients; the second is based on cadaveric surgery on three specimens. The average IRD reduction after TP in the study was 0.6 cm and 0.7 cm for the two defined measurement points (representing 26% and 28% of the preoperative IRD, respectively). The reduction in IRD after TP was statistically significant. The mean IRD reduction after TP in the three cadaveric specimens was 0.7 cm (25% of the preoperative IRD). If DRA is defined as 2 cm at any IRD point, TP abdominoplasty can correct DRA when the preoperative inter-recti distance is less than 2.5 cm, or between 2.5 and 3 cm in the absence of visceral fat obesity. In conclusion, although TP abdominoplasty reduces IRD, we recommend longitudinal plication abdominoplasty (either isolated or combined with transverse plication) when the IRD exceeds 3 cm, is between 2.5 and 3 cm in the presence of visceral fat obesity, or when complete approximation of the medial edges of the rectus abdominis is desired.

## Introduction

Diastasis recti abdominis (DRA) is defined as a condition characterized by excessive separation—generally considered to be 2 cm or more—between the medial edges of the rectus abdominis muscles along the linea alba (LA), which may result in abdominal wall dysfunction. In isolated DRA, there is no disruption of the abdominal wall layers, but the loss of strength and the presence of midline laxity can facilitate the development of associated hernias.^1^^,^^2^

DRA is rarely congenital and is more commonly observed after pregnancy or significant weight loss. Symptoms of isolated DRA are usually cosmetic in nature, often involving dissatisfaction with body image. As for non-cosmetic symptoms, patients may report weakness in abdominal tone and strength, lower back pain, or symptoms related to pelvic floor muscle weakness.^3^, ^4^, ^5^ However, the causal relationship between DRA and these conditions remains a matter of debate, as they are multifactorial and variable in nature, making it difficult to distinguish causation from mere association.^1^

When a patient presents with symptoms suggestive of DRA, various strategies can be employed to evaluate the inter-recti distance (IRD) and confirm the diagnosis. Palpation and external measurement may serve as initial assessments of DRA severity, but accurate imaging—such as ultrasound, CT, or MRI—is necessary to precisely quantify the condition. Ultrasound evaluation of IRD is a safe, simple, and rapid method for obtaining accurate measurements at various points along the LA.^6^ According to the criteria established by Beer et al., the threshold for diagnosing DRA is an IRD greater than 22 mm, measured by ultrasound 3 cm above the umbilicus.^7^

Non-surgical treatment based on physiotherapy should be the first-line therapeutic approach.^2^^,^^8^^,^^9^ If DRA persists after 6–12 months of conservative treatment, and the patient experiences significant aesthetic or functional discomfort, surgical intervention may be considered.^3^, ^4^, ^5^ Vertical or longitudinal plication (LP) of the aponeurosis at the LA is widely regarded as the gold standard surgical technique for most cases of isolated DRA.^2^^,^^10^, ^11^, ^12^, ^13^, ^14^

During abdominoplasty, other types of anterior aponeurosis plication have been described, either in conjunction with or independent from LP.^15^, ^16^, ^17^, ^18^, ^19^ Horizontal or transverse plication (TP) of the infraumbilical anterior aponeurosis is likely the second most common technique after LP to enhance abdominal wall contour.^20^ In recent years, the TULUA abdominoplasty—described by Dr. Villegas and based on TP without flap undermining—has gained popularity due to its ability to achieve excellent contouring results while reducing complications such as skin necrosis, hematoma, seroma, and wound healing or scar-related complications.^18^^,^^21^, ^22^, ^23^

Nevertheless, the effect of TP on DRA remains controversial, as diastasis is not directly addressed in this technique. It has been hypothesized that shortening and tightening of the anterior aponeurosis may contribute to a reduction in IRD. Imaging studies using MRI have shown a decrease in IRD in some isolated cases following TP.^21^ The purpose of this study is to evaluate the effect of TP on IRD and, consequently, its capacity to treat DRA.

## Materials and methods

Two different strategies were designed to investigate whether TP can reduce IRD and effectively treat DRA. The first is a prospective self-controlled case series study with ultrasound evaluation; the second is based on cadaveric specimen dissection.

### Self-controlled case series study

The study was conducted in accordance with the STROBE guidelines, and the protocol received approval from the Research Ethics Committee of the Hospital Clínic of Barcelona. Patients were recruited consecutively from those referred to the Plastic Surgery Department of Hospital Clínic de Barcelona with a history of bariatric surgery and subsequent massive weight loss, presenting with excess abdominal skin. Following clinical examination at the outpatient clinic, IRD was measured via ultrasound. If the IRD exceeded 1.5 cm at any point and no exclusion criteria were identified (Table 1), patients were invited to participate in the study. The study's purpose was explained in detail to each patient, and informed consent for both participation and surgery was obtained.

**Table 1:** Exclusion criteria.

Exclusion criteria	
1	Previous abdominoplasty.
2	Previous surgery to correct DRA.
3	Previous major surgeries of abdominal wall defect: eventrations or hernias that required correction with meshes.
4	Presence of eventrations or hernias without previous treatment.
5	DRA greater than 4.5 cm.

If surgical correction of abdominal skin excess was indicated but the patient declined participation or met exclusion criteria, abdominoplasty was offered outside the study. If the IRD was greater than 4.5 cm, abdominoplasty with LP was indicated.

**Figure 1:**
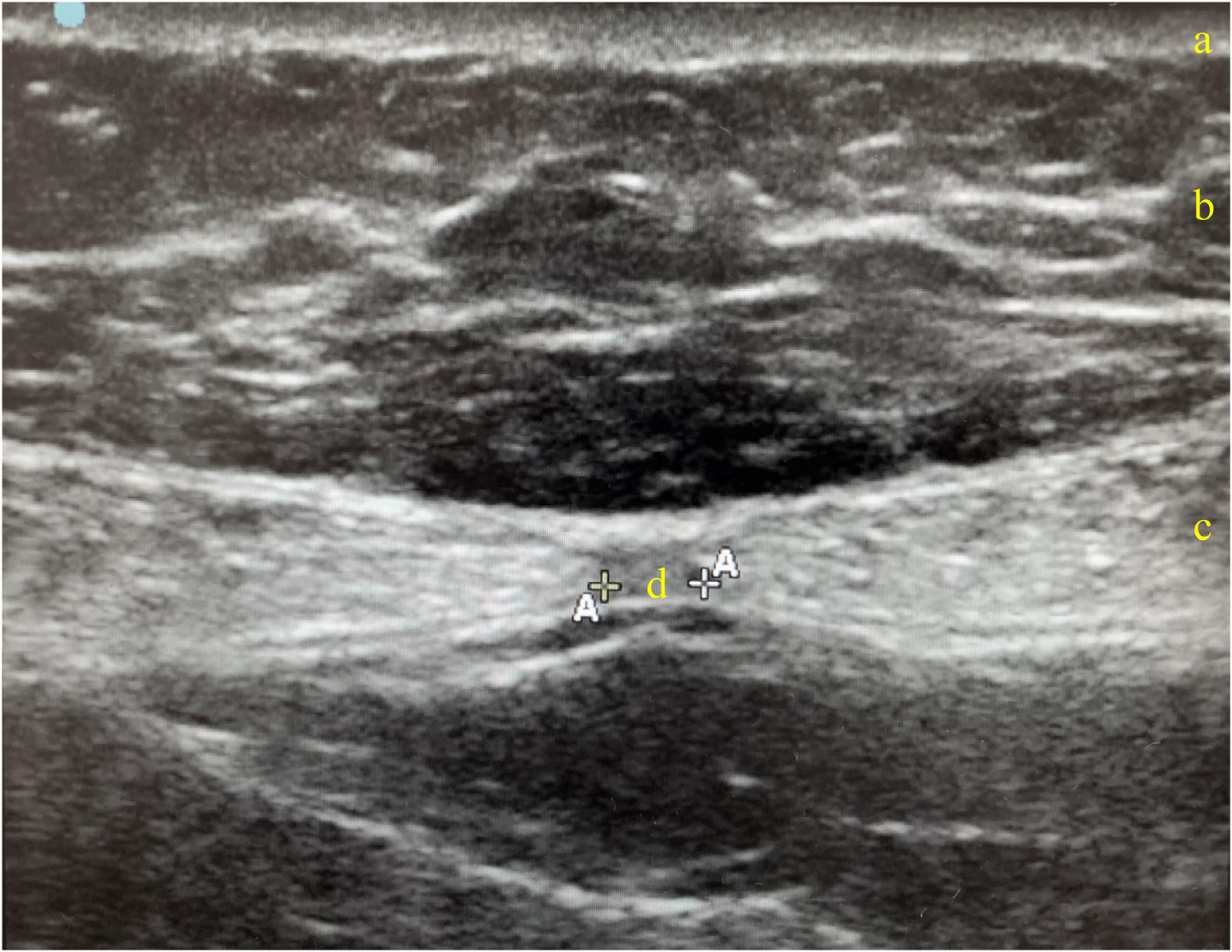
Ultrasound measurement of the IRD. (a) Dermis layer; (b) Subcutaneous tissue layer; (c) Rectus abdominis muscles layer; (d) IRD measurement, defined as the distance between the medial edges of the rectus abdominis muscles: A landmarks visible on the screen.

**Figure 2:**
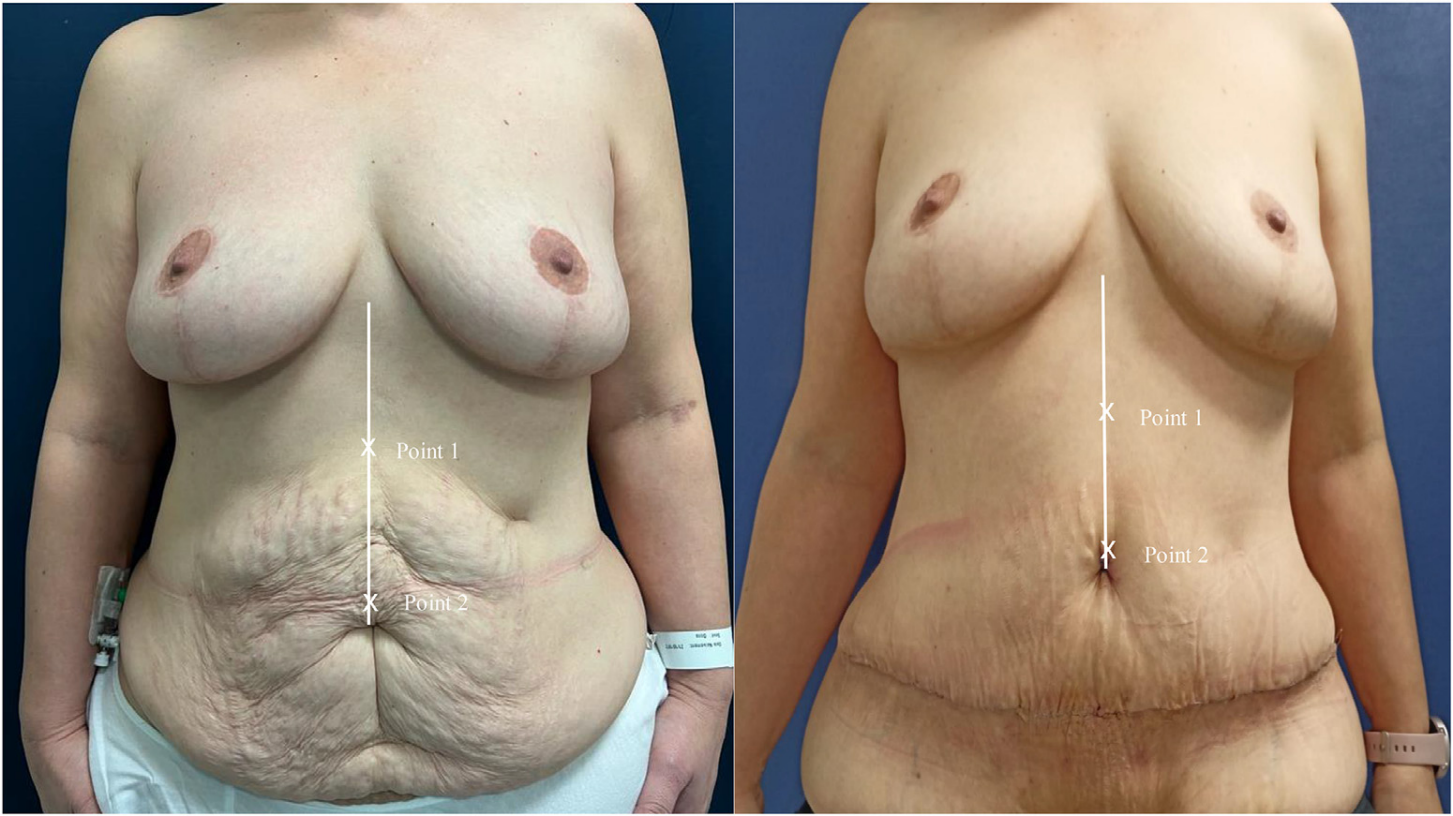
Preoperative and postoperative frontal views of a patient included in the study. Preoperative ultrasound measurement points were defined according to: Point 1, the midpoint between the xiphoid process and the umbilicus; and Point 2, the level of the umbilicus. Postoperative ultrasound measurement points were defined according to: Point 1, the midpoint between the xiphoid process and the location of the neoumbilicus; and Point 2, the level of the neoumbilicus.

Sixteen consecutive post-bariatric patients for whom TP abdominoplasty was indicated and who consented to participate were included in the study. Preoperative IRD was assessed using ultrasound (SonoSite SII with the linear 13-6 MHz transducer), measuring the distance between the medial edges of the rectus abdominis muscles (Figure 1) at two anatomical landmarks: Point 1 (midpoint between the xiphoid process and the umbilicus) and Point 2 (umbilicus). Postoperative IRD measurements were taken again at both points at 1 week, 1 month, 3 months, 6 months, and 1 year following surgery. Since the original umbilical position was no longer available after the surgery due to neoumbilicoplasty, postoperative Points 1 and 2 were defined in relation to the new umbilicus location (Figure 2).

The primary variable was the ultrasound-measured IRD (in cm). Secondary variables were categorized into patient-dependent and surgery-dependent variables (Table 2).

**Table 2:** Secondary variables.

Secondary variables	
Patient dependent	Surgery dependent
Sex, age (years); weight (kg); height (m); BMI (kg/m^2^); number of previous pregnancies; complications at delivery; previous conservative treatment of DRA; active smoking	Resection flap weight (kg), Postoperative pain (VAS) during admission, hemoglobin reduction after surgery, days of admission, transfusion needed, hematoma, seroma, wound healing complications, scar complications

Descriptive and inferential statistical analyses were performed using SPSS version 25 for Windows. In the descriptive analysis, IRD reduction at Points 1 and 2 during follow-up visits was assessed. In patients with a preoperative IRD >2 cm, correction of DRA was defined as a postoperative IRD <2 cm. Inferential statistics were used to determine whether the reduction in IRD following TP was statistically significant.

### Surgical technique for recruited patients

Preoperative markings were performed with the patient in a standing position. The inferior incision was placed 6 cm above the anterior commissure of the labia majora (or the root of the penis in male patients). The superior incision was marked above the umbilicus. Lateral extension of markings was made as needed to avoid dog ears.

Liposuction was not performed, as the primary goal was functional restoration after massive weight loss rather than cosmetic improvement. The marked skin and subcutaneous tissue were excised, including division of the umbilical stalk. No undermining of the supraumbilical tissue was performed. The operating table was set at 30°, and the elliptical TP was marked on the anterior aponeurosis of the infraumbilical wall.

**Figure 3:**
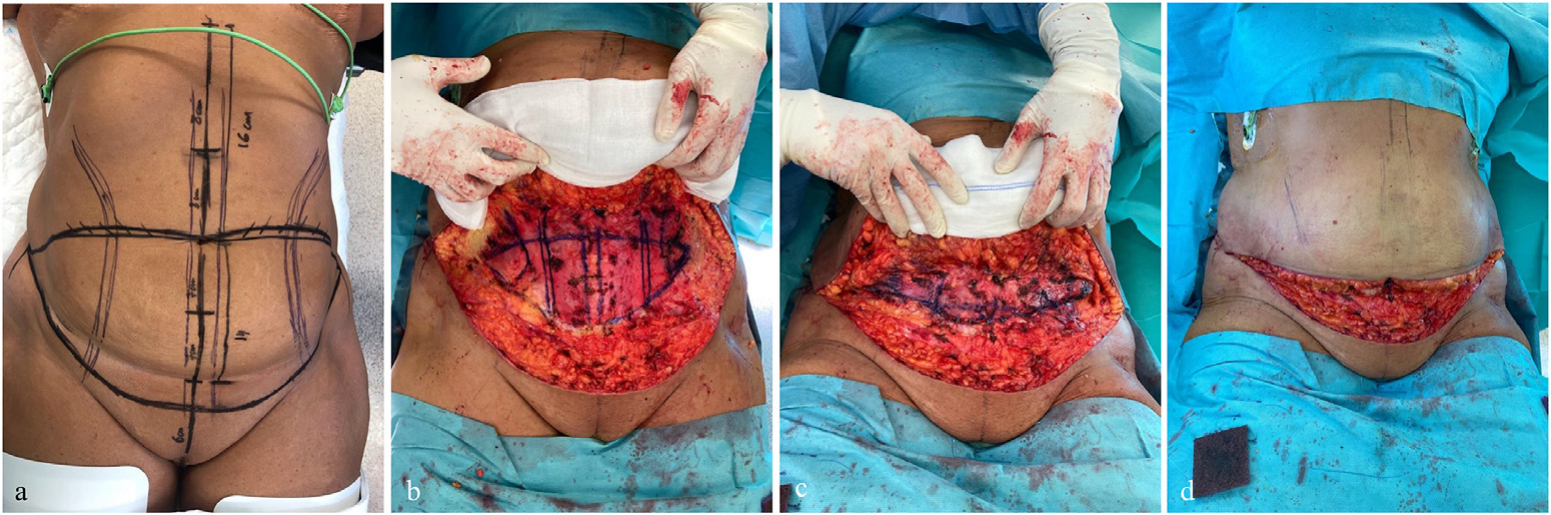
Surgical technique. (a) Preoperative markings: the inferior incision was placed 6 cm above the anterior commissure of the labia, and the superior incision above the umbilicus. (b) TP was planned and marked above the anterior aponeurosis after excision of the skin and subcutaneous tissue, including division of the umbilical stalk. (c) TP was performed using inverted horizontal mattress 2 Ti-Cron sutures. (d) Skin could be closed without tension and without undermining the supraumbilical tissue.

Following the TP technique described by Villegas et al. in the TULUA procedure, TP size was determined using the “four-finger depression” maneuver to assess wall laxity.^24^ TP was performed using inverted horizontal mattress 2-Ti-Cron (braided polyester) sutures. After suturing Scarpa’s fascia and closing the skin under minimal tension, neoumbilicoplasty was performed at the midline. The position of the neoumbilicus above the scar was measured using a sterile ruler and determined based on golden ratio from the anterior vulvar commissure or penile root to the scar.^18^ Neoumbilicoplasty was performed using 3-0 and 4-0 nylon sutures, either with an H-flap technique or a full-thickness skin graft from the excised tissue, depending on the midline subcutaneous thickness.^18^^,^^21^^,^^22^^,^^24^^,^^25^ Two size-12 Redon drains were placed in all cases (Figure 3).

### Surgical intervention on cadaveric specimens

**Figure 4:**
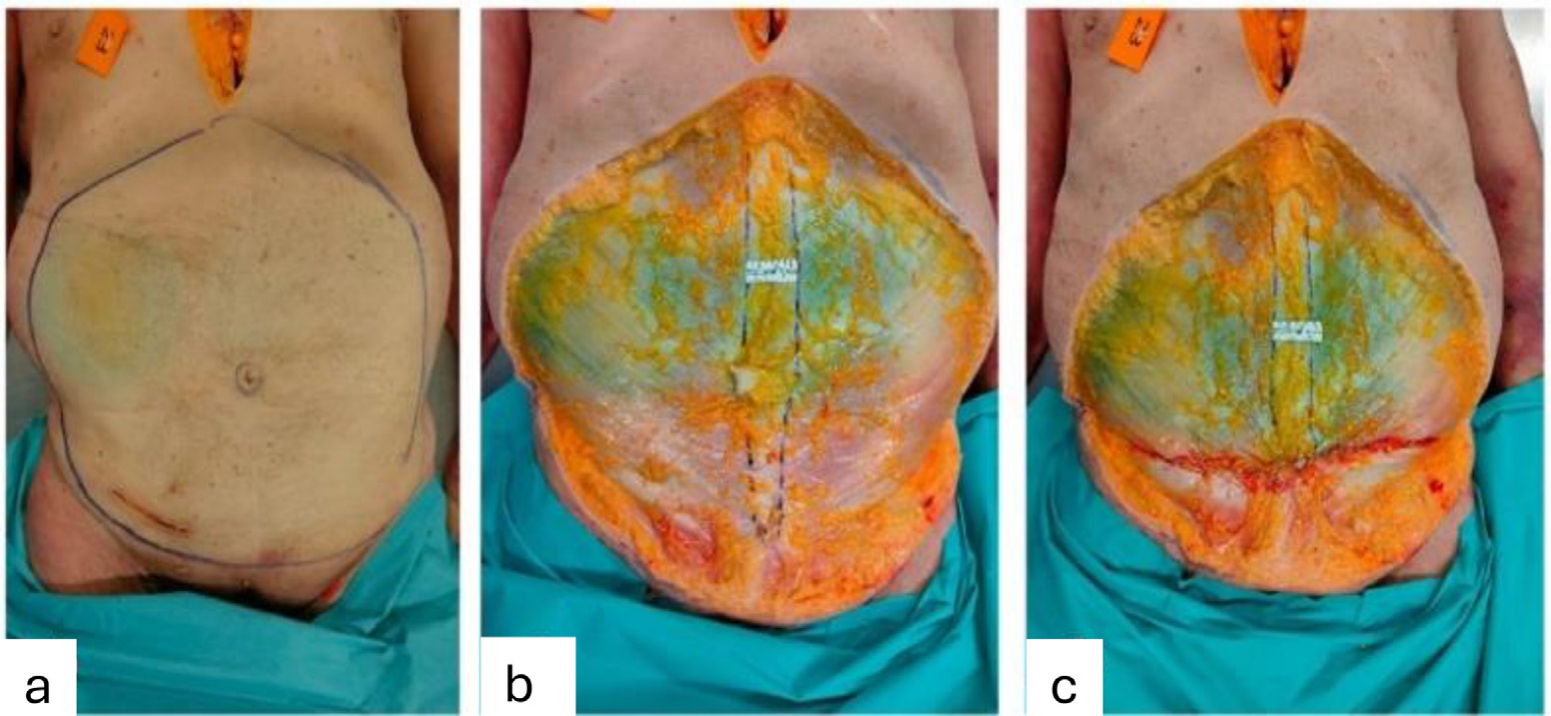
Surgical intervention on the second cadaveric specimen. (a) Marking of the skin and subcutaneous tissue to be excised. (b) Exposure of the anterior aponeurosis throughout the entire abdomen, allowing a direct measurement of the IRD of 3,2 cm at Point 1 (the midpoint between the xiphoid process and the umbilicus) prior to TP. (c) Measurement of the IRD at the same point after TP showed a reduction to 2.5 cm.

TP was performed on three cadaveric specimens following removal of the abdominal skin and subcutaneous tissue. The specimens were provided by the Department of Anatomy at the University of Barcelona, and the procedure was carried out in the anatomy laboratory of the Universitat de Barcelona Campus Clínic. IRD was measured at Point 1 before TP and again after the procedure at the same location. Since the entire anterior abdominal aponeurosis was exposed, changes in IRD following TP could be directly visualized and measured at the exact same point, offering precise assessment of TP’s effect on DRA. Point 2 was not evaluated, as the upper limit of the TP extended above the umbilicus and couldn’t be measured after TP (Figure 4).

## Results

### Self-controlled case series study

Three male and thirteen female patients, aged between 25 and 62 years (mean age 50 ± 8.7), were included in the study. All patients had a history of bariatric surgery and had experienced significant weight loss. The mean BMI prior to TP abdominoplasty was 28.4 ± 2.2. Only patients 6 and 9 reported being active smokers and continued smoking despite strong recommendations to quit prior to surgery. Eight of the thirteen female patients had a history of pregnancy, with an average of 1.5 pregnancies among them. Three patients had undergone previous conservative treatment for DRA during the first year postpartum (Table 3).

**Table 3:** Selected secondary variables

	Patient dependent					Surgery dependent					
Patient	Sex (F/M)	Age (years)	BMI (kg/m2)	Previous Pregnancies	Delivery complications	Flap Weight (kg)	Pain VAS	Hb reduction (g/dL)	Admission days	Transfusion needed	Complications
1	F	53	27,4	1	NO	1,6	2	0,8	2	NO	NO
2	F	54	28,1	0	-	1,9	5	1,4	3	NO	NO
3	F	25	29,8	0	-	2,1	4	0,9	2	NO	NO
4	F	49	29,3	2	1 Caesarean	2,4	6	2	2	NO	NO
5	F	59	28,7	1	1 Caesarean	2,2	6	1,3	3	NO	NO
6	M	42	28,7	-	-	3,6	4	1,2	2	NO	Seroma
7	F	45	29,6	1	NO	1,7	7	2	3	NO	NO
8	F	48	26	1	NO	1,3	4	4,8	4	1 RBC	Hematoma
9	F	49	25,4	3	3 Caesarean	1,5	5	0,7	2	NO	NO
10	F	62	29,7	0	-	2,7	5	1,6	3	NO	NO
11	M	44	26,5	-	-	2,2	3	1,1	2	NO	NO
12	M	51	27,5	-	-	3,1	5	1,4	3	NO	NO
13	F	39	32,8	2	NO	2,8	8	1	3	NO	NO
14	F	56	29,7	0	-	2,8	3	0,9	2	NO	NO
15	F	59	23,7	0	-	1,1	5	1,6	2	NO	NO
16	F	48	31,6	1	NO	2,9	5	2	3	NO	NO

Following surgery, the weight of resected abdominal skin and subcutaneous tissue ranged from 1.1 kg to 3.6 kg, with a mean of 2.2 ± 0.7 kg. The mean pain score on the Visual Analog Scale (VAS), assessed during hospitalization, was 4.8 ± 1.5. Patients remained hospitalized for an average of 2.6 ± 0.6 days. The mean postoperative hemoglobin (Hb) reduction was 1.5 ± 0.9 g/dL. Only patient 8 required one unit of red blood cell concentrate (RBC) due to a drop in Hb of 4.8 g/dL, primarily attributed to a mild postoperative hematoma. This complication was managed conservatively with compression and ultrasound-guided percutaneous drainage during outpatient follow-up. Patient 6 developed a postoperative seroma of 175 cc, which was resolved with compression and two ultrasound-guided drainages (Table 3).

Regarding the primary study variable, the preoperative IRD at Point 1 ranged from 1.2 cm to 3.8 cm (mean 2.3 ± 0.8 cm), and at Point 2 from 1.6 cm to 3.9 cm (mean 2.5 ± 0.7 cm). All patients had at least one measurement (Point 1 or Point 2) greater than 2 cm—associated with potential DRA symptoms—except patients 2, 3, 6, 8, and 11, in whom both values were below 2 cm (Table 4).

**Table 4:** Average of the IRD reduction after TP (in absolute values and percentage of the preoperative IRD) from the measures obtained at each follow-up visit during the first year for each patient.

Patient	Preoperative measures (cm)		pO DRA	Average reduction in absolute values (cm) and percentage		PO DRA
	Point 1	Point 2		Point 1	Point 2	
1	2,8	2,7	P	1,0 (36%)	0,8 (30%)	A
2	1,6	1,7	A	0,3 (19%)	0,3 (18%)	A
3	1,7	1,8	A	0,6 (35%)	0,7 (39%)	A
4	**1,2**	2,3	P	0,2 (17%)	1,1 (48%)	A
5	1,7	2,3	P	0,5 (29%)	0,4 (16%)	A
6	1,5	**1,6**	A	0,3 (20%)	0,3 (19%)	A
7	2,7	2,9	P	1,7 (63%)	1,7 (59%)	A
8	1,4	1,7	A	0,4 (29%)	0,2 (12%)	A
9	**3,8**	**3,9**	P	0,8 (21%)	0,8 (21%)	**P**
10	3,4	3,8	P	0,7 (21%)	1,0 (26%)	**P**
11	1,9	1,7	A	0,5 (26%)	0,2 (12%)	A
12	2,1	2,3	P	0,7 (33%)	1,1 (48%)	A
13	3,8	3,4	P	0,3 (8%)	0,2 (6%)	**P**
14	2,1	2,3	P	1,1 (52%)	1,3 (57%)	A
15	2,3	2,6	P	0,5 (22%)	0,9 (35%)	A
16	2,9	2,8	P	0,1 (3%)	0,2 (7%)	**P**
**X̅**	**2,3**	**2,5**	**11 P**	**0,6 ± 0,39** **(26% ± 17%)**	**0,7 ± 0,45** **(28% ± 18%)**	**4 P**

Presence (P) or absence (A) of preoperative (pO) DRA—at least one measure above 2 cm—and presence or absence of postoperative (PO) DRA.

Postoperative IRD measurements at the predefined follow-up visits showed a mean reduction of 0.6 ± 0.39 cm at Point 1 (ranging from 0 cm to 1,9 cm) and 0.7 ± 0.45 cm at Point 2 (ranging from 0 cm to 1,9 cm). These absolute reductions represented, on average, a 26 ± 17% decrease at Point 1 and a 28 ± 18% decrease at Point 2 compared to preoperative values. Four of the eleven patients with preoperative DRA continued presenting IRD values >2 cm after TP (Table 4).

IRD measurements during the 1-year follow-up remained mostly stable. The average IRD reductions recorded at each follow-up visit were consistent with the overall mean reductions: 0.6 cm at Point 1 and 0.7 cm at Point 2. Only the 1-month postoperative assessment at Point 2 showed a slightly higher reduction of 0.8 cm.

The Shapiro–Wilk test confirmed a normal distribution of the data. Therefore, Student’s t-test was used for hypothesis testing to assess the statistical significance of IRD reduction following TP. The test yielded p-values < 0.05 for both Point 1 and Point 2, indicating that the reduction in IRD after TP was statistically significant.

### Surgical intervention on cadaveric specimens

The three cadaveric specimens included two females and one male, aged between 50 and 70 years. None had signs of prior abdominal surgeries, and no additional medical history was available.

Direct IRD measurements at Point 1 before TP were 3.1 cm, 3.2 cm, and 2.1 cm. After TP, the same points measured 2.0 cm, 2.5 cm, and 1.8 cm, respectively (Table 5 and Figure 4).

**Table 5:** Point 1 IRD of the three cadaveric specimens before and after TP. IRD reduction (in absolute values and percentage of the preoperative IRD).

Specimen	Sex (F/M)	Preoperative measures (cm)	Postoperative measures (cm)	IRD reduction (cm) and percentage
		Point 1	Point 1	Point 1
1	F	3,1	2,0	1,1 (35%)
2	F	3,2	2,5	0,7 (22%)
3	M	2,1	1,8	0,3 (14%)
**X̅**		2,8	2,1	0,7 (25%)

## Discussion

Transverse plication (TP) techniques in abdominoplasty have been widely described in the literature, either as isolated procedures or in combination with other anterior aponeurotic plications.^17^ Since the introduction of the TULUA technique by Villegas et al.^21^, TP has gained increased attention among plastic surgeons performing abdominoplasty. The hallmark of this approach is the execution of a broad TP without flap undermining. The entire preservation of the vascular and sensory integrity of the superior flap and its complete attachment provides a plausible pathophysiological explanation for the high safety profile reported with the TULUA procedure. For surgeons who do not routinely use TULUA, it may be particularly indicated in patients with extensive previous abdominal scars or when significant liposuction is required.

While complication rate analysis was not an objective of this case series—and a larger sample would be necessary to assess it adequately—only two patients in the study experienced minor postoperative complications, both managed successfully without revision surgery. Patient 8 developed a mild hematoma, which was treated conservatively. The absence of flap undermining and the resulting limited potential space for hematoma formation may have contributed to the effectiveness of compression therapy in achieving hemostasis. Patient 6 developed a postoperative seroma, potentially related to active smoking. Similarly, the resolution of the seroma following two ultrasound-guided percutaneous drainages may have been favoured by the lack of a large undermined area where fluid could accumulate.

Although the TULUA technique includes liposuction as part of the procedure, no liposuction was performed in any of the patients in this study. All patients were referred to the plastic surgery department to address the functional sequelae of massive weight loss. Within the context of Spain's national healthcare system, which supports reconstructive rather than cosmetic interventions, liposuction is generally not indicated for these patients. Nonetheless, the absence of liposuction did not interfere with the main objective of the study or the evaluation of the primary variable. In fact, it facilitated more accurate postoperative ultrasound assessment of IRD, as recent liposuction could have compromised visualization, particularly for the deeper to the subcutaneous layer.

The impact of TP on IRD and DRA remains a subject of debate. Critics of TP argue that decreasing the compliance of the inferior anterior aponeurosis could increase IRD above the plication site, potentially contributing to epigastric bulging. Conversely, proponents suggest that the reduction in anterior aponeurosis length and the increase in tension may lead to secondary IRD reduction.^17^ This has been observed in retrospective case reports using MRI.^21^ However, most surgeons who incorporate TP in their practice still advocate for LP—either as a standalone technique or in combination with TP through a limited tunnel undermining above DRA—when treating patients with significant DRA. The precise IRD threshold for recommending LP lacks consensus among these surgeons and remains unvalidated.^26^^,^^27^

To date, the effect of TP on IRD has not been evaluated systematically in a prospective study. This self-controlled case series is the first study specifically designed to assess the impact of TP on IRD and its potential role in the treatment of DRA.

Although sample size of the study is relatively small (16 patients), the power of the study comes from the fact each patient serves as their own control (preoperative IRD) eliminating the need for a separate control group. In addition, the TP surgical intervention performed on three cadaveric specimens yielded results consistent with those of the clinical study. Although cadaveric tissues may not fully replicate in vivo biomechanics, the concordant findings in the specimens support the results of the self-controlled case series and may reduce the need for a larger study sample.

The IRD threshold inclusion criterion was set at 1.5 cm instead of 2 cm, as the primary objective was to identify IRD variations rather than to assess DRA treatment. The DRA threshold in the study was set at 2 cm, in line with routine considerations in clinical practice. The DRA diagnostic criteria established by Beer et al.^7^ (IRD greater than 22 mm, 3 cm above the umbilicus) could only be applied before TP, since the original umbilical reference is completely altered after surgery. Study reference points 1 and 2 were defined in relation to the original umbilicus preoperatively, and to the neoumbilicoplasty positioning after TP abdominoplasty. In cadaveric TP, point 1 could be determined prior to TP, and the IRD could be measured at the exact same location after TP, as complete exposure of the anterior aponeurosis eliminated the need to identify points relative to anatomical landmarks. Although no specific threshold had been previously validated for LP indication, an IRD greater than 4.5 cm was considered an exclusion criterion, as such severe DRA could objectively benefit from LP procedure during abdominoplasty.

None of the patients in the study showed an increase in IRD after TP. However, some patients—particularly those with a high preoperative IRD and minimal reduction—reported a subjective sensation of epigastric bulging, which typically improved progressively during the first year following surgery. The specific effect of TP plication on epigastric bulging, independent of IRD, is currently being analysed as a second part of this study.

IRD reductions in the study ranged from 0 cm to 1.9 cm, with an average reduction of 0.6 cm at point 1 and 0.7 cm at point 2, and reduction in IRD after TP was statistically significant. These results are consistent with those obtained in the three cadaveric TP cases, where the average IRD reduction was 0.7 cm. Analysis of the variation in IRD reduction between patients revealed that the lowest reductions were observed when the preoperative IRD was greater than 3 cm or when significant visceral fat was present. Visceral fat measurement—whether by waist circumference, waist-to-hip ratio, waist-to-height ratio or ultrasonography^28^—should have been reported as a secondary, patient-dependent variable. This could have helped to quantify visceral fat and provide a more detailed analysis of its relationship with the limited IRD reduction following TP. Although it has been demonstrated that significant IRD reductions can be achieved with TP, the predictability for individual patients remains a major concern when DRA is expected to be treated using this technique.

Of the 11 patients presenting with preoperative DRA, only four had an IRD >2 cm after TP. Three of them (patients 9, 10, and 13) were the only ones with a preoperative IRD >3 cm. The other patient (patient 16) had a preoperative IRD slightly below 3 cm, but presented with significant visceral fat. Therefore, if DRA treatment is the goal, we recommend LP—either combined with TP or alone—when the preoperative IRD is greater than 3 cm, or when it is between 2.5 cm and 3 cm and significant visceral fat is present. When the IRD is below 2.5 cm, DRA is more likely to be successfully treated with TP alone.

It is also important to highlight that, following TP, the IRD very rarely falls below 1 cm. The smallest IRD measured after TP was 0.8 cm (at point 1 in patient 7, at the 1-month postoperative follow-up). Therefore, even when achieving a significant reduction in IRD after TP, a minimal physiological separation of the medial edges of the rectus abdominis muscles should be expected—unlike with LP, where complete approximation of the rectus muscles is routinely pursued.

IRD reductions following TP have been shown to remain largely stable during the first year after surgery. Minor variations in IRD measurements at the different follow-up visits may be attributed to the measurement procedure, slight changes in body weight, or variations in abdominal content. As no significant or consistent differences were observed throughout the 1-year follow-up, obtaining multiple measurements for each patient helped to identify the true mean reduction and mitigate measurement variability.

## Conclusion

TP abdominoplasty is a safe procedure that may be especially indicated when the risk of postoperative complications is higher than usual. When properly indicated, TP can reduce the IRD by more than 0.5 cm. However, it can be difficult to predict the exact reduction that will be achieved in a specific patient. Regarding DRA, TP abdominoplasty can correct diastasis when the preoperative IRD is less than 2,5 cm, or between 2,5 and 3 cm in the absence of visceral fat obesity. LP (either isolated or combined with transverse plication) should be considered when the IRD exceeds 3 cm, or when complete approximation of the medial edges of the rectus abdominis is desired.

## Conflict of interest statement

None of the authors have any disclosure regarding financial and personal relationships with other people or organizations that could inappropriately influence (bias) this work.

## Funding

None.

## Ethical approval

The study protocol was approved by the Research Ethics Committee of Hospital Clínic de Barcelona at its meeting held on October 21, 2021 (Minutes of Meeting No. 18/2021).

## Ethical statement

Ethics approval for this study was obtained from the Research Ethics Committee of the Hospital Clínic of Barcelona, as documented in minutes 18/2021 of the meeting held on 21 October 2021.

## Declaration of AI and AI-assisted technologies in the writing process

During the preparation of this work the authors used ChatGPT in order to improve readability and language, not to replace key researcher tasks such as interpreting data or drawing scientific conclusions. After using this tool the autors reviewed and edited the content as needed and take full responsibility for the content of the publication.

## Declaration of competing interest

None declared.
